# Vasoactive intestinal peptide exerts therapeutic action by regulating PTEN in a model of Sjögren's disease

**DOI:** 10.1002/iid3.936

**Published:** 2023-07-12

**Authors:** Yixi Li, Wen Zhu, Rui Lin, Junjie Zhao, Yue Wang

**Affiliations:** ^1^ Nanjing University of Chinese Medicine The First School of Clinical Medicine Nanjing China; ^2^ Jiangsu Province Hospital of Chinese Medicine Affiliated Hospital of Nanjing University of Chinese Medicine Nanjing China; ^3^ Nanjing University of Chinese Medicine School of Health and Rehabilitation, Jiangsu Key Laboratory of Respiratory Disease, Institute of Pesdiatrics, Medical Metabolomics Center Nanjing China

**Keywords:** PI3K/AKT, PTEN, Sjögren's disease, Treg/Th17, vasoactive intestinal peptide

## Abstract

**Introduction:**

Sjögren's disease (SjD) is a chronic autoimmune disease characterized by the loss of the secretory function of the exocrine glands. At present, drugs that can both correct the immune imbalance and improve exocrine gland function are needed. Meanwhile, vasoactive intestinal peptide (VIP) has been reported as a candidate with anti‐inflammatory and immunoregulatory properties for treating autoimmune diseases.

**Methods:**

Nonobese diabetic (NOD) mice and the primary splenic lymphocyte cells (SPLCs) were used to construct the SS model. The therapeutic effects of VIP for SjD by evaluating water consumption, histopathology, T cell subsets, and related cytokines. RT‐qPCR and Western blot analysis were used to identify the expression of the PTEN/PI3K/AKT pathway.

**Results:**

We found that VIP therapy in NOD mice could increase the expression of PTEN and VIP/VPAC1 receptor, as well as decrease the PI3K/AKT pathway. In vitro, the results showed that the PTEN knockdown decreased the Treg/Th17 ratio and enhanced the phosphorylated PI3K/AKT pathway, which were reversed with VIP treatment.

**Conclusions:**

VIP exerts potential therapeutic action in SjD by upregulating PTEN through the PI3K/AKT pathway and Treg/Th17 cell balance.

## Graphical abstract

Vasoactive intestinal peptide (VIP) might be effective for the treatment of Sjögren disease. VIP exerts potential therapeutic action in Sjögren's disease by upregulating PTEN, acting partly through the PI3K/AKT pathway and Treg/Th17 cell imbalance.

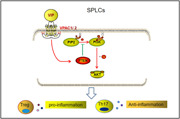

## 1 INTRODUCTION

Sjögren's disease (SjD), also known as Sjögren's syndrome (SS), occurs in primary and secondary conditions defined by the impaired secretory function of the exocrine glands, especially the salivary and lacrimal glands.^1^, ^2^ According to epidemiological survey, the global prevalence of SS is 1 case per 1644 persons, and the female incidence rate is higher than the male.^3^ The pathological feature of SS is the infiltration of lymphocytes in affected organs and tissue, as well as the increased levels of positive auto‐antibodies.^4^ Although the mechanism of this disease remains unclear, SS has been classically considered a disease of B cells on the basis of evidence that B cell activating factor (BAFF) is continuously detected in the damaged tissue. Then transition into the more recent research that has identified the participation of T‐cell subsets in this process.^5^ Several functional CD4+ T‐cell subsets, including helper T 17 (Th17) cells and regulatory T (Treg) cells, modulate immunity activation by production of pro‐inflammatory cytokines plus interaction with B cells. Treg cells are identified as a small subset of immune cells which can suppress the immune system and maintain immune homeostasis.^6^, ^7^ Genetic alteration resulting in a decrease in Treg cells can exacerbate sialadenitis. The study supports the idea that Treg cells play a prime representative in preventing autoimmunity of the salivary glands in healthy individuals.^8^ Since the suppressive effects of Treg cells on some autoimmune diseases, Treg cells can confer tissue repair against the damaging effects during some stages of pSS.^9^ Moreover, numerous studies have indicated that Th17 cells can contribute to the disease pathology of mouse models in the affected tissues.^10^, ^11^

As a prosecretory and vasodilating neuropeptide, the vasoactive intestinal peptide (VIP) has potent immunomodulatory effects on T cells.^12^ Local gene therapy using an adenoviral construct encoding VIP could repair the salivary function in the model of SS.^13^ Our former research found that treating with VIP in an SS model could reduce the immune injury of IL‐17A to exocrine glands.^14^ However, the molecular mechanism by which VIP signaling regulates the differentiation and function of T cells is still elucidated.^15^ Phosphatase and Tensin homolog on chromosome 10 (PTEN), a tumor suppressor protein with dual specific protein and phospholipid phosphatase activity, can express ubiquitously and mediates cellular processes such as cell survival and apoptosis.^16^ Mechanistically, PTEN maintains Treg cell stability and the metabolic balance between glycolysis and mitochondrial fitness.^17^ Besides, PTEN acts as an antagonist of the phosphoinositide 3′ kinase (PI3K)/AKT pathway, thereby regulating the immune system.^18^ The PI3K/AKT signaling pathway plays a pivotal role in balancing the Th17/Treg ratio and cell proliferation.^19^ Both VIP and PTEN play an essential role in maintaining immune homeostasis. But whether there is a synergistic effect between the two is still unknown. In this study, we hypothesized that PTEN might involve in the underlining mechanisms of VIP in treating SS. The role of VIP in the regulation of the immune status and secretory function of salivary glands was examined in NOD mice and C57BL/6 mice. VIP and si‐RNA targeting PTEN were used to intervene in splenic lymphocyte cells (SPLCs), and the intervention effect of VIP on the PTEN/PI3K/Akt pathway and the balance of Treg/Th17 was investigated.

## 2 MATERIALS AND METHODS

### 2.1 Animals

Eighteen female nonobese diabetic (NOD) mice and seven female C57BL/6 mice were accommodated in a specific pathogen‐free facility. All mice were 8 weeks old and were raised under standard conditions (12 h light‐dark cycle, 25–27°C, 40% humidity) with adequate water and food. At first, these samples acclimated for 1 week. A total of 21 mice with a group of seven mice were assigned. The NOD mice were divided randomly into a model group and a VIP therapy group, and the C57BL/6 mice were used as controls. The therapy group was given 1.5 nmol VIP (synthesized by China Peptides; purity ≥98%) via intraperitoneal (IP) injection every other day. The mice in the control and the model group were given pure water by IP injection. After VIP intervention for 8 weeks, mice were killed under an anesthetized state. The rest four NOD mice were used to isolate splenic lymphocyte cells (SPLCs) for preparation.

### 2.2 Water intake, saliva flow rate, and histological analyses

The method of water intake and saliva flow rate were measured as published.^20^ Saliva production was stimulated at a dose of 0.5 mg/kg pilocarpine (Yuanye Bio‐Technology) through intraperitoneal injection. Water consumption was recorded weekly from Week 1 to Week 8, and the saliva flow rate was determined on Week 8. After removing and fixing with 4% paraformaldehyde in PBS, the submandibular glands (SMGs) were embedded in paraffin. Next, standard hematoxylin‐eosin (H&E) staining was then directed on rehydrated paraffin segments. With the help of the BX43 microscope (Olympus), the Chisholm and Mason classification assessed the degree of lymphocytic infiltration of submandibular tissue.^21^ PTEN, PI3K, and p‐PI3K in SMGs were in immunohistochemical detection. Frozen tissue sections were embedded in optimum cutting temperature (OCT) compound and sectioned. After that, the tissues were incubated with anti‐PTEN (ab267787; Abcam), anti‐PI3 Kinase p85 alpha (ab225720; Abcam), anti‐VIP (16233‐1‐AP; Proteintech), and after washes with goat anti‐rabbit MaxVision Kit (MXB Biotechnologies), followed by DAB color reaction. Finally, the sections were counterstained with hematoxylin before dehydration and then cover‐slipped. The brown staining represented a positive area. Using Image J software to define the pixels threshold to distinguish region of interest from background, the average optical density was measured by the percentage of the integrated optical density to the positive staining.

### 2.3 Enzyme‐linked immunosorbent assay (ELISA) and quantitative polymerase chain reaction (qPCR)

Spleen tissue was minced in PBS containing 1 mM PMSF and centrifuged at 12,000 g for 20 min at 4°C. The expression of IL‐2 and IL‐10 was measured using the Mouse IL‐2 and Mouse IL‐10 ELISA kit (both from MultiSciences) according to the manufacturer's instructions. Serum extraction method as published,^20^ VIP levels in serum were measured using the mouse VIP ELISA kit (Elabscience). For qPCR analyses, total RNA from the spleen was individually isolated using Total *RNA* Extraction Reagent (Vazyme), and *cDNAs* were obtained using Hifair® II 1st‐Strand *cDNA* Synthesis SuperMix (Yeason). Further quantitative analysis of genes was done by Hieff® qPCR SYBR Green Master Mix (Yeason) in a two‐step reaction and performed using the QuantStudio 7 Flex (Thermo Fisher Scientific). Each sample was tested in triplicate, and the 2^−ΔΔ^CT cycle threshold method was used for the analysis of relative gene expression. The sequences of qPCR primers are listed in Table 1 Primers sequences used for gene transcription levels determination.

**Table 1:** Primers sequences used for gene transcription levels determination.

Gene name	Sequence (5′–3′)
PTEN‐F	AGTTTGTGGTCTGCCAGCTAA
PTEN‐R	AGGTTTCCTCTGGTCCTGGTA
PI3K‐F	CGAGACGGCACTTTCCTTGT
PI3K‐R	CGGTGGCAGTCTTGTTAATGAC
AKT‐F	GCCGCCTGATCAAGTTCTCC
AKT‐R	GGCTTCTGGACTCGGCAATG
VIP‐F	AGTGTGCTGTTCTCTCAGTCG
VIP‐R	GCCATTTTCTGCTAAGGGATTCT
VPAC1‐F	GATGTGGGACAACCTCACCTG
VPAC1‐R	GATGTGGGACAACCTCACCTG
VPAC2‐F	GACCTGCTACTGCTGGTTG
VPAC2‐R	CAGCTCTGCACATTTTGTCTCT
GAPDH‐F	AGCCCAAGATGCCCTTCAGT
GAPDH‐R	CCGTGTTCCTACCCCCAATG

### 2.4 SPLCs isolation and transfection

SPLCs were isolated from the spleen tissue of NOD mice (17‐week‐old) under sterile conditions. The tissues were in a 70 μm cell strainer and were gently pressed with the plunger seal of a 1‐mL syringe. Briefly, the spleen tissue was mechanically digested, filtered, and further purified by density gradient centrifugation using the Mouse Splenic Lymphocyte Isolation Kit (Solarbio Life Sciences). The splenic lymphocyte suspension was cultured with RPMI1640 (Thermo Fisher Scientific) and supplemented with 10% fetal bovine serum (FBS) (Gibco). PTEN was selectively knocked down in the SPLCs, using the synthesized siRNA targeting PTEN (namely, mpten931): 5′‐CGACUUAGACUUGACCUAUAUTT‐3′ (sense) and 5′‐AUAUAGGUCAAGUCUAAGUCGTT‐3′ (antisense) (Shanghai Sangon Biological Engineering Co., Ltd.). Mpten931 was transfected into cells by the RNATransMate reagent (Shanghai Sangon Biological Engineering Co., Ltd.).

### 2.5 Cell‐counting kit‐8 assay and Western blot assay

A total of 5 × 10^3^ cells/well of each group were seeded into 96‐well plates (Corning), cultured at 37°C for 12 h with 5% CO_2_, and then 10 μl CCK‐8 solution (Beyotime) was added to each well. The cells were then cultured at 37°C with 5% CO_2_ for another hour. Then the value of optical density (OD) in each group was evaluated 450 nM using Microplate ReaderM200 PRO (Thermo Fisher Scientific). For western blot analysis, the following antibodies were used: Phospho‐pan‐AKT (Ser473; Affinity), Phospho‐PTEN (S380; Affinity), GAPDH (AF7021; Affinity), anti‐PI3K (ab191606; Abcam) and anti‐AKT (ab18785; Abcam).

### 2.6 Flow cytometry

The SPLCs were collected into FACs tubes and washed in PBS. Before staining‐stimulated SPLCs for analysis, restimulated lymphocytes were treated with 5 μg/mL brefeldin A (Biovision, C) for 6 h. Treg cells were stained with AlexaFluor 488‐anti‐CD4, PE‐cyanine7‐anti‐CD25, and stained further with PE‐anti‐Foxp3. Th17 cells were stained with AlexaFluor 488‐anti‐CD4 and AlexaFluor 647‐ anti‐IL‐17A (eBioscience). Acquisitions were performed using the Navios flow cytometer (Beckman Coulter). Foxp3 Transcription Factor Staining Buffer Set Kit (eBioscience) was used in fix and permeabilize cells. FlowJo10 software was used to measure the percentage of CD25+ /FOXP3+ Tregs and IL‐17+ Th17 cells in the CD4+ gate.

### 2.7 Statistical analysis

The Shapiro–Wilk normality and the KS normality test were used to check Gaussian‐ distribution of the data, all at alpha >0.05. Unpaired student *t*‐test (two‐tailed) was used to analyze the differences between the two experimental groups. One‐way analysis of variance (ANOVA) with Tukey′s multiple‐comparisons test was used for comparing means between groups considering only one independent factor. All analyses were acquired with GraphPad Prism 8.2.1 using *p* < 0.05 as statistically significant. The results are expressed as the means ± standard deviation (SD). Each experiment was repeated at least three times.

## 3 RESULTS

### 3.1 Exogenous VIP relieved lymphocytic infiltration in the SMGs

**Figure 1:**
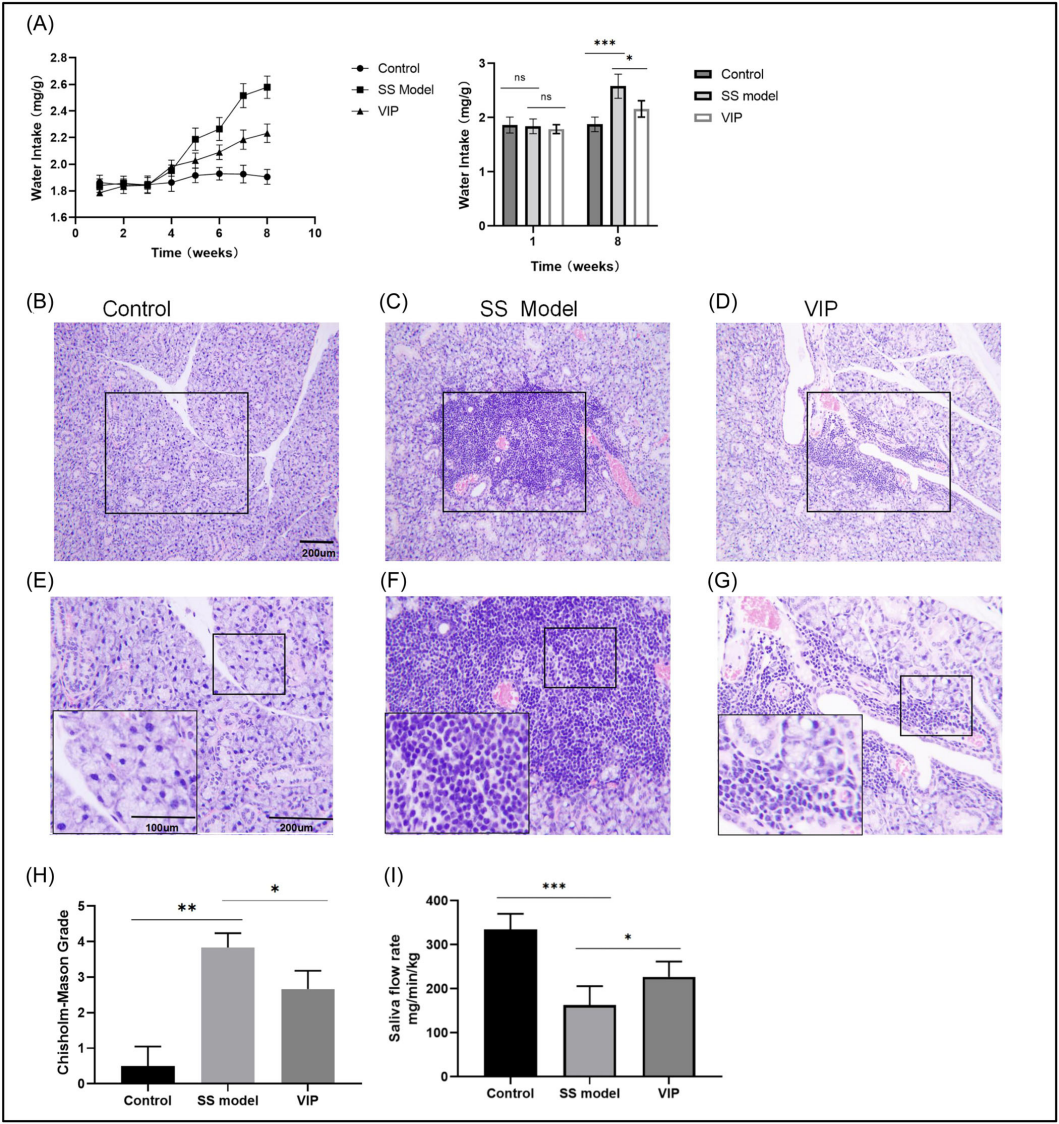
Vasoactive intestinal peptide (VIP) relieved lymphocytic infiltration in the submandibular glands (SMGs) of Sjögren's syndrome (SS) mice. (A) The water intake of the Control, SS model, and VIP groups were calculated on a weekly basis (*n* = 7). (B–G) SMG tissues were examined using hematoxylin‐eosin (H&E) stained and observed under the 10x objective (B–D) and 20x objective (E–G). The lower left corner in (E–G) is a magnified panel for the black rectangle in the figure. (H) The Chisholm–Mason grade was determined. (I) Saliva flow rates were determined after receiving intervention for 8 weeks (*n* = 7). Scale bar: 200 µm. *p < 0.05, **p < 0.01, ***p < 0.001.

Since the development and pathogenesis of NOD mice could be defined by the spontaneous infiltration of salivary gland lymphocytes under dry oral conditions.^22^ We chose the NOD mice whose sialadenitis in the SMGs as the SS mice. C57BL/6 mice, known to be resistant to autoimmune diseases, were selected as the control group.^23^ The average water consumption of the model and VIP groups was elevated starting around 5 weeks and continued till 8 weeks. But only 8 weeks after intervention, the water intake of the VIP therapy group was significantly lower compared with the SS model group (*p* < 0.05) (Figure 1A). As shown in Figure 1B–G, the lymphocytic infiltration and formed lymphocytic foci were seen in the model of SS group. In contrast, the SMG from the control group was represented as a normal salivary gland. Lymphocytic infiltration was mild in the VIP group. The Chisholm–Mason grade was applied to assess the lymphocytic infiltration (Figure 1H). Mice receiving VIP therapy, had a better overall histological condition than those from the model group (*p* < 0.05). Consistently, compared to the mice from model group in Figure 1I, more salivary flow rate was seen than those in the VIP group (*p* < 0.05). These results indicated that exogenous VIP could effectively ameliorate sialadenitis with dry mouth in the SMGs of SS mice.

### 3.2 Exogenous VIP increased PTEN and VIP expression in the SMGs of SS mice

**Figure 2:**
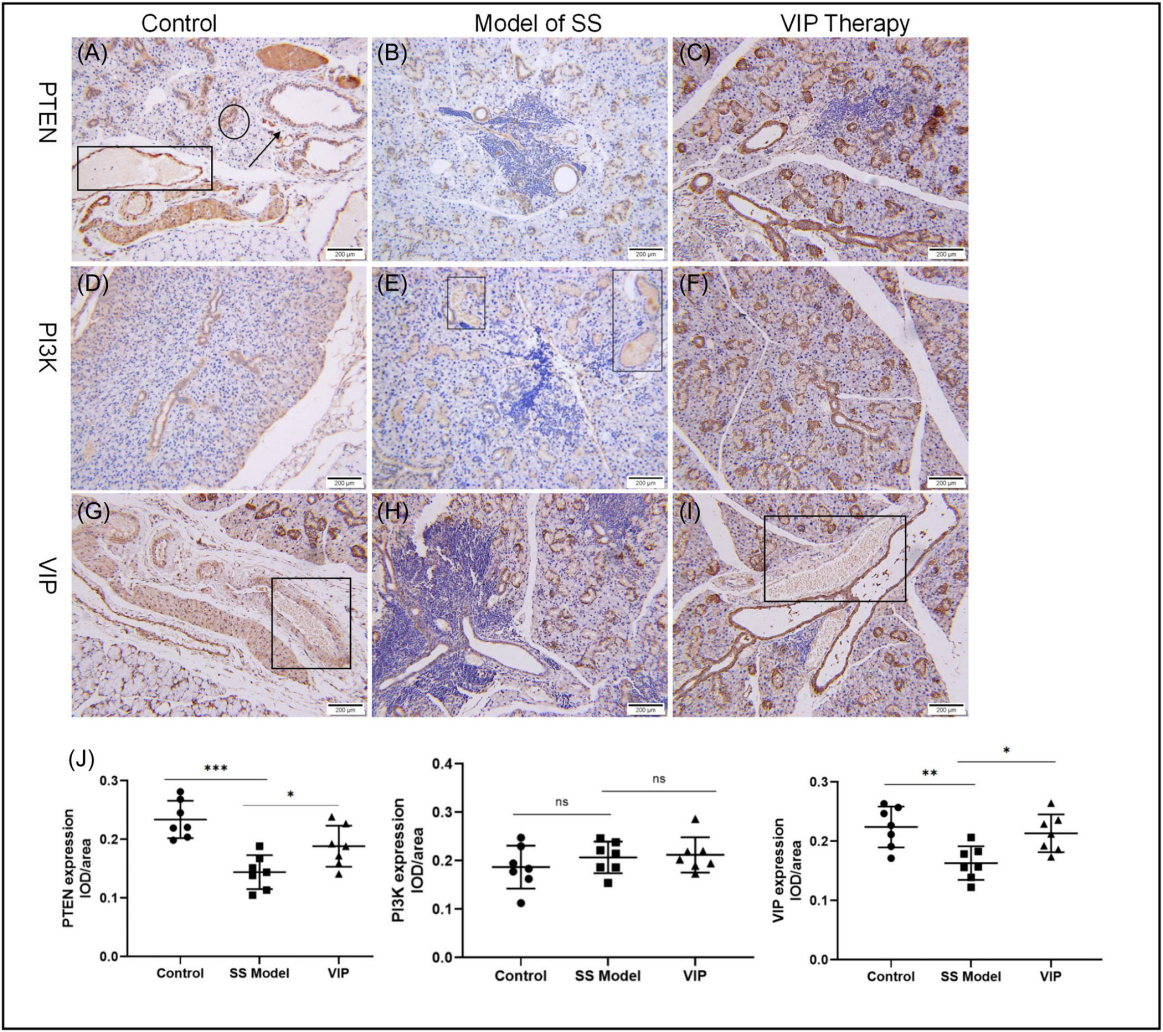
Immunohistochemical staining was performed to label PTEN, PI3K, and vasoactive intestinal peptide (VIP) in submandibular glands (SMGs). PTEN (A‐C), PI3K (D‐F), and VIP (G‐I) are present in SMGs of the control group, Sjögren's syndrome (SS) model group, and VIP therapy group. Rectangle = serous acini; Circle = acinar cells; Arrow = ductal cells. Scale bar: 200 µm. (J). Quantitative analyses of PTEN, PI3K, and VIP expression in the SMGs (*n* = 7). IOD, integrated optical density.

As shown in Figure 2A,C, PTEN protein expression was detected in the ductal cells of and the serous acini of the control group and VIP group but not in the model of SS group (Figure 2B). In contrast, detectable PI3K immunostaining was in the serous acini of the model of SS group and VIP group (Figures 2E,F) but not in the control mice (Figure 2D). The decreased saliva secretion and atrophy of the salivary gland are associated with pathological changes of SS. This might explain why we do not observe the expression of PTEN in the serous acini of the SS group. Similarly, numerous VIP positive staining surrounding a ductal cell was seen in the control group and VIP therapy group (Figures 2G,I) but not in the model of SS group (Figure 2H). Overall, PTEN and VIP expression in the SMGs was significantly increased after VIP treatment (Figure 2J).

### 3.3 VIP regulated PTEN/PI3K/AKT pathway, VIP/VIP receptor signaling, and anti‐inflammatory cytokines in the spleen of SS mice

**Figure 3:**
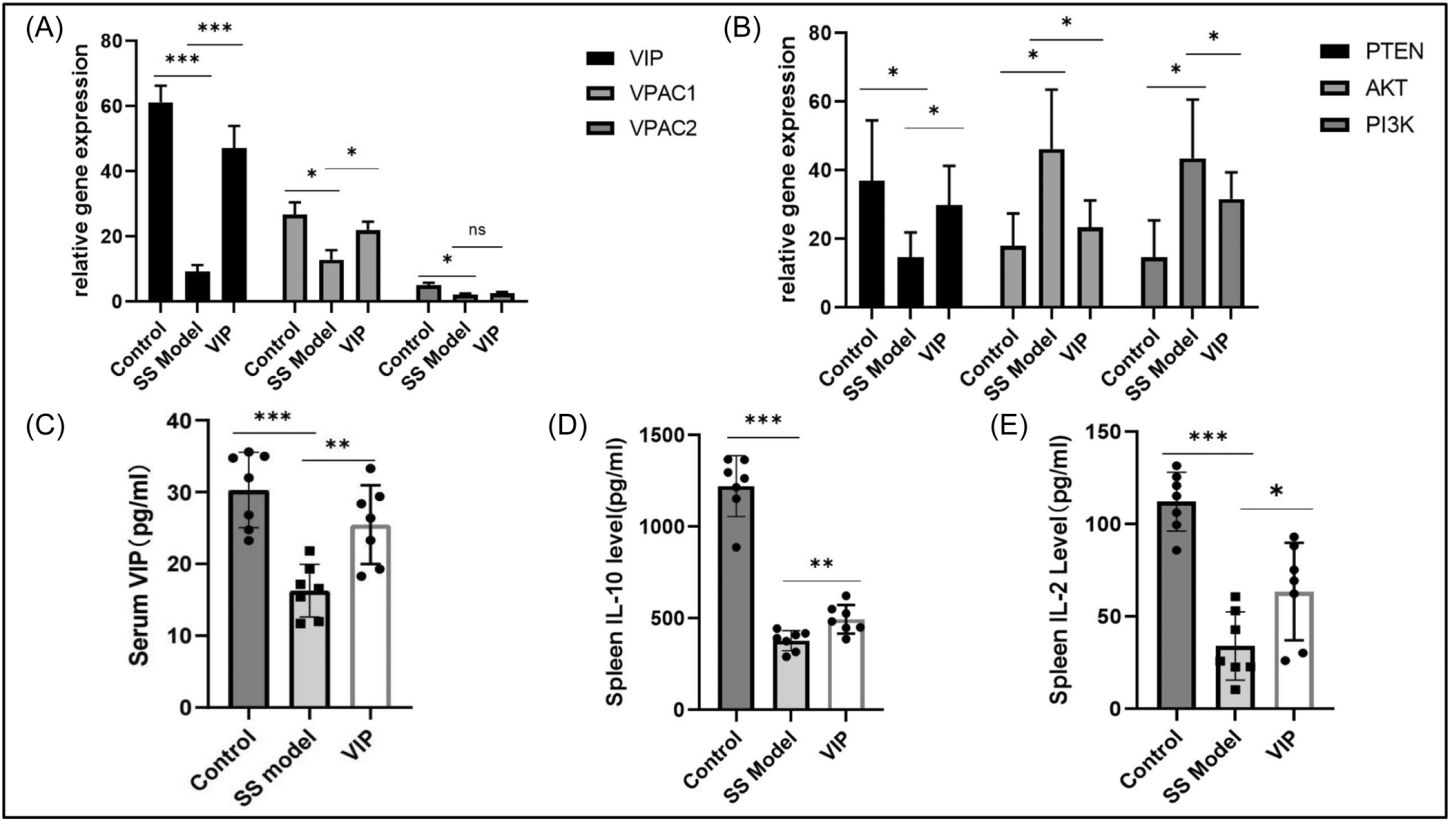
Vasoactive intestinal peptide (VIP) upregulated PTEN, VIP receptor mRNA expression, and Treg‐related cytokines in spleen. Q‐PCR results for VIP mRNA, VIP receptors mRNA expression (A), and PTEN/PI3K/AKT pathway (B) in the spleen. The concentrations of VIP in the serum (C) were measured using ELISA kits (*n* = 7). The abundance of interleukin (IL)‐10 (D) and IL‐2 (E) in the splenic tissue homogenate supernatant was determined by ELISA as well (*n* = 7). Unpaired Student *t*‐test was used in the analysis. *p < 0.05, **p < 0.01, ***p < 0.001.

The VIP and VIP receptors signaling, as an immunomodulatory factor, may play a protective role in the pathogenesis of autoimmune diseases. PTEN negatively regulates the PI3K/AKT pathway, and the mRNA of the PTEN/PI3K/Akt pathway was determined in the spleens from these groups. In comparison to the model group, the expression of PTEN, VIP, and VPAC1 mRNA was increased in the VIP group (Figure 3A,B) (*p* < 0.05). Conversely, the expression of PI3K and AKT mRNA from VIP and the control groups was significantly decreased than those in the SS mice (Figure 3B) (*p* < 0.05). As shown in Figure 3C, the serum levels of VIP in SS mice were significantly lower than those in the control and VIP groups (*p* < 0.05). IL‐10 is a wide spectrum of anti‐inflammatory activity, and IL‐2 performs therapeutic ability in treating autoimmune diseases. The levels of IL‐2 and IL‐10 in the spleen tissue homogenates from SS mice were significantly lower than those in the control and VIP group (Figure 3D–E) (*p* < 0.05). These results indicated that exogenous VIP regulated PTEN/PI3K/AKT pathway, VIP/VIP receptor signaling, and related Treg cytokines in the spleen of SS Mice.

### 3.4 VIP increased the expression of phosphorylated‐PTEN and decreased the phosphorylated PI3K/AKT pathway

**Figure 4:**
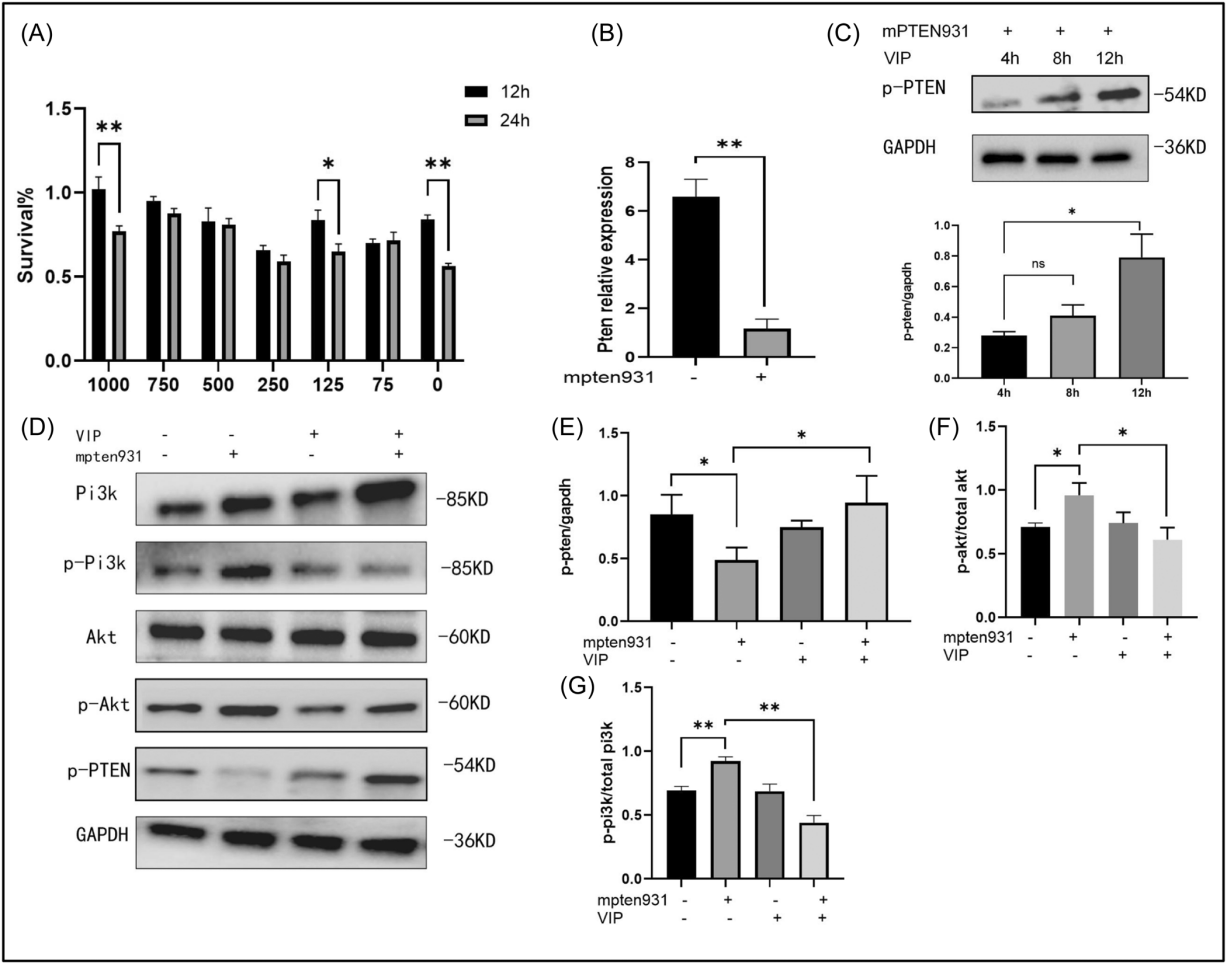
The effect of vasoactive intestinal peptide (VIP) on the phosphorylation of PTEN and PI3K/AKT in Splenic lymphocyte cells (SPLCs). (A) SPLCs were isolated from the nonobese diabetic (NOD) mice and plated for 12 or 24 h with different doses of VIP. A CCK‐8 assay was used to detect the optical density (OD) value of each group. The data were analyzed by a mixed‐effects model approach with Geisser‐Greenhouse correction. Tukey's multiple comparisons post hoc test was conducted following analysis of variance (ANOVA). (B) SPLCs were transfected with 5 pM mpten931 (siRNA targeting PTEN). At 24 h post‐transfection, the knockdown efficiency of mpten931 was determined by qRT‐PCR. (C) Cells were treated with 125 ng/μL VIP for the indicated times at 24 h post‐transfection. The data were analyzed with one‐way ANOVA. (D) Representative WB experiments. Cells were transfected or not with mpten931 followed by treatment with 0 or 125 ng/μL VIP for 12 h. (E–G) Protein expression levels of total AKT, phosphorylated AKT, total PI3K, phosphorylated PI3K, and phosphorylated PTEN were analyzed by Western immunoblotting (WB). Cumulative data from three independent experiments. *p < 0.05, **p < 0.01.

Primary SPLCs were cultured in a complete medium for 24 h. Then CCK‐8 assay was used to measure the cell viability after the inducement of different concentrations of VIP inducement (1000, 750, 500, 250, 125, 62.5, and 0 ng/μL) 12 h or 24 h (Figure 4A). According to the results of CCK‐8, the cell viability after treating 12 h was higher than those in treating 24 h for this screening. Additionally, a dose‐dependent splenic lymphocyte proliferation caused by VIP was not seen. Then, at concentrations of 125 ng/μL and treating for 12 h were chosen in the following experiments. As shown in Figure 4B, mpten931 was successfully transfected into SPLCs. VIP time‐dependently increased the expression of phosphorylated‐PTEN after silencing PTEN with mpten931 (Figure 4C). As shown in Figure 4D–G, the results showed that PTEN knockdown decreased phosphorylated‐PTEN and increased p‐PI3K/total PI3K and p‐AKT/total AKT ratio in SPLCs. However, decreased expressions of PI3K/AKT pathway by VIP were not observed in SPLC cells. Nevertheless, reduced phosphorylation of PTEN and increased p‐PI3K/total PI3K, and p‐AKT/total AKT ratio by mpten931 were abolished in the treatment with VIP.

### 3.5 VIP regulated Treg/Th17 cell balance via PTEN pathway

**Figure 5:**
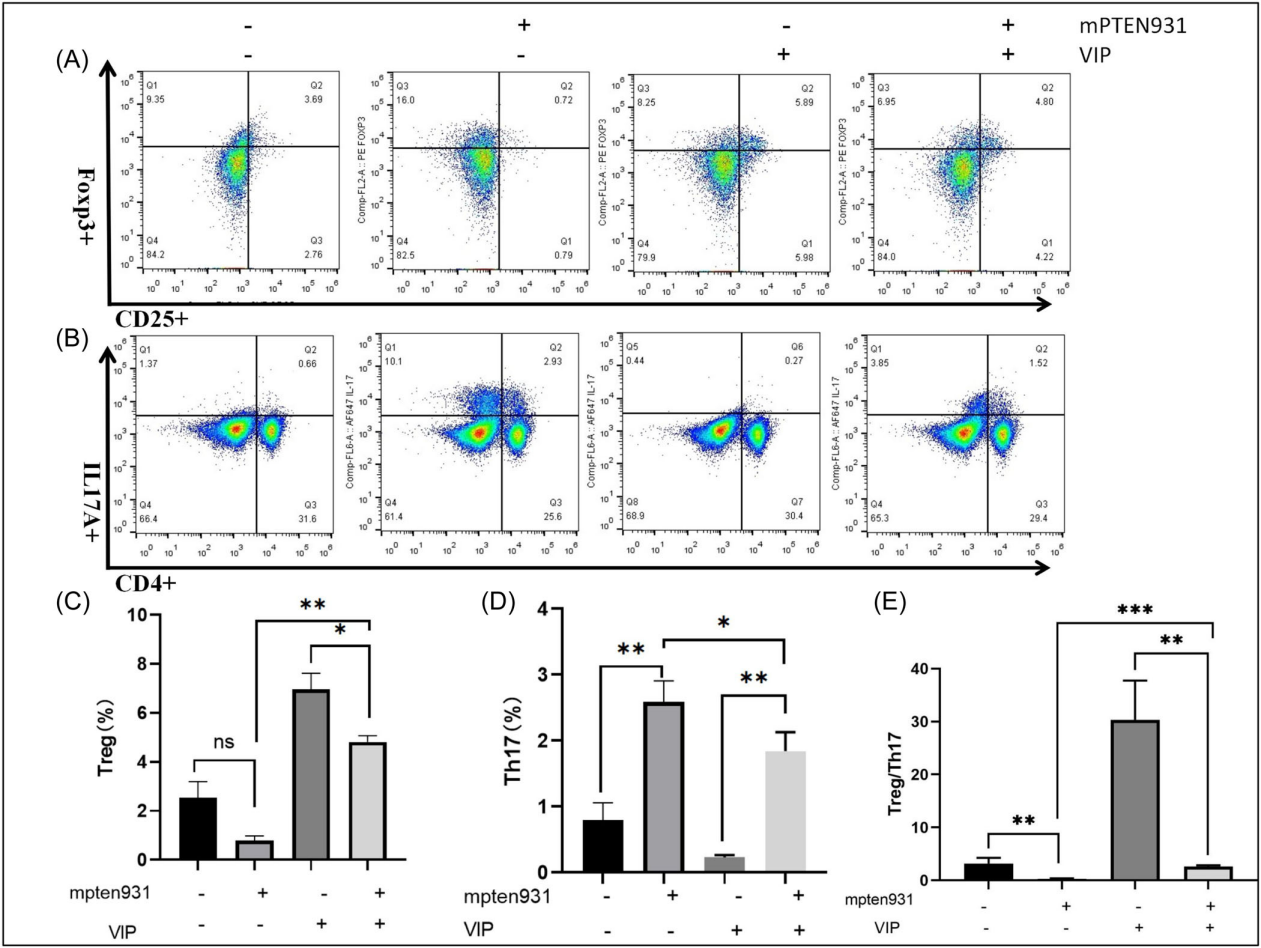
The proportions of Treg and Th17 cells: The CD4+ events are shown on the plots. After splenic lymphocytes transfection, representative flow cytometry analysis of Foxp3+ CD25+ CD4+ Treg (A) and IL17A+ CD4+ Th17 (B) in ex vivo culture with 0 or 125 ng/μL vasoactive intestinal peptide (VIP) for 12 h. The frequency of Treg cells (C), Th17 (D), and the ratio of Treg/Th17 (E). *p < 0.05, **p < 0.01, ***p < 0.001.

The proportion of Treg and Th17 cells in the SPLCs was measured by flow cytometry (Figure 5A,B). The Treg cells were identified as CD4+ CD25+ Foxp3+, while Th17 cells were identified as CD4+ IL17A+. As shown in Figure 5C,D, the results showed an increase of the proportion of Th17 cells and a decrease of the proportion of Treg cells in the SPLCs with mpten931. However, treatment of VIP decreased the frequency of Th17 cells, and increased the frequency of Treg cells (Figure 5E). Our results indicated that PTEN knockdown decreased the ratio of Treg/Th17 cells, which was reversed with VIP treatment.

## 4 DISCUSSION

In this study, we used NOD mice as the SS model and administered with VIP via intraperitoneal injection. The lymphocytic infiltration and an increased number of water consumption, as well as a decreased saliva rate, have been observed in the NOD mice. These results were basically consistent with the previous study about the SS model of mice.^24^

VIP is considered an anti‐inflammatory mediator that has multiple biological characteristics. It has been defined that VIP is involved in decelerating the progression of autoimmunity diseases, such as rheumatoid arthritis and osteoarthritis.^25^ However, few studies investigate the therapeutic mechanism of VIP in treating SS. Here the alleviating effect of exogenous VIP was identified by clinical symptoms, histopathology, and anti‐inflammatory cytokines. IL‐2 and IL‐10 are related to cytokines in the anti‐inflammation and functional Treg cells. Moreover, high‐affinity IL‐2 receptors are constitutively expressed on Treg cells, making these cell populations very sensitive.^26^ Additionally, several studies have shown that VIP caused a progressive protection in the sialadenitis of NOD mice and upregulated the anti‐inflammatory factors.^27^, ^28^ Researchers have previously reported that VIP improved the secretory function and reduced the expression of IL‐17A in a model of SS.^14^ Thus, we did not specifically investigate the effects of VIP on the expression of IL‐17A in NOD mice. Here, we revealed that exogenous VIP upregulated the levels of IL‐2 and IL‐10, as well as alleviated sialadenitis with dry mouth. These findings suggested that VIP may play the therapeutic role of SS. However, a broader panel of cytokines is still ongoing to investigate by which VIP altered in treating SS in the future work, such as interferon‐γ and BAFF. The underlying mechanisms of VIP treatment in the disease are complex, and more evidence is needed.

SS is commonly known to be correlated with lymphomas,^29^ which is the most severe complication associated with local chronic inflammatory stimulation of B cells. B cell lymphoma in SS frequently occurs in salivary glands, whereas PTEN suppresses tumor cell growth and survival. Soypacaci et al.^30^ have reported that PTEN protein is expressed in 87.2% of SS outpatients in the salivary gland in a retrospective evaluation. However, the disadvantage of this research is the absence of a healthy control group. Here, our results indicated that PTEN has lower expression in salivary glands from the SS mice than those in the healthy group. Additionally, VIP therapy promoted the expression of PTEN in vivo and in vitro. Thus, the possibility of a synergistic effect exists between VIP and PTEN. Notably, PTEN antagonizes the PI3K‐AKT pathway, which is a classic pro‐inflammatory signaling pathway. The PI3K/AKT pathway enhances the levels of pro‐inflammatory cytokines and chemokines, as well as regulates immune activities. In this study, inhibition of the PI3K/AKT pathway and increased VIP receptor VPAC1 by VIP intervention were observed in NOD mice. It agrees with a previous study that the activation VIP‐receptor system decreases AKT activation.^31^

**Figure 6:**
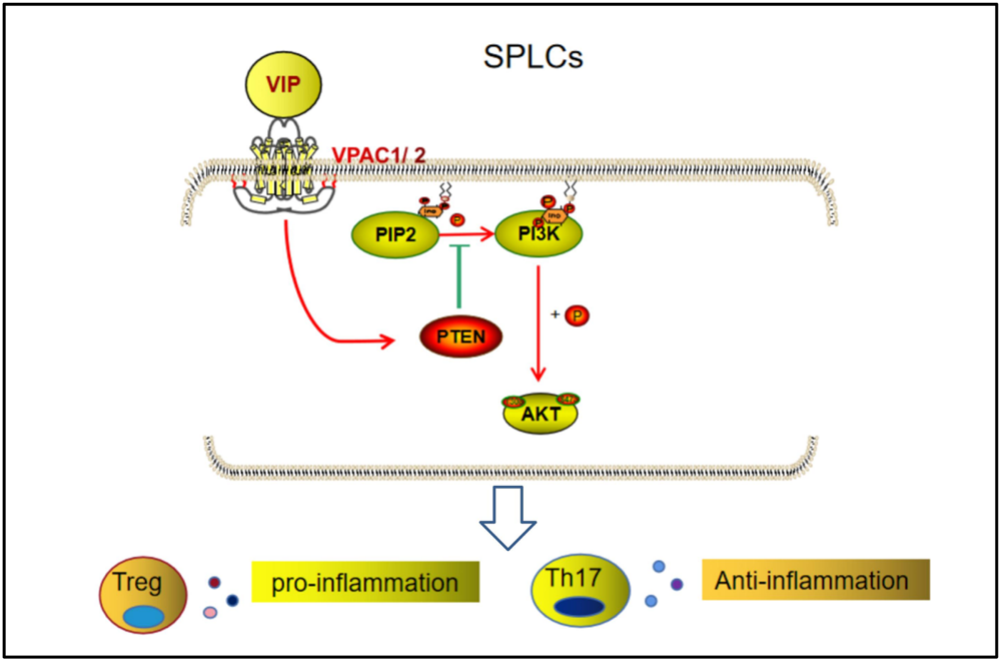
The schematic figure incorporating the signaling pathways. The red arrow represents promotion; the green arrow represents inhibition.

Interestingly, accumulating data have demonstrated that activation of the PI3K/AKT pathway is involved in modulating the T cell subsets, including the balance of Treg/Th17 cells.^32^, ^33^ The function and differentiation of T cells are precisely, thus avoiding the impairment of normal organizations. In abnormal conditions, T‐cell activation can induce inflammation and local damage. Therefore, the role of T cell suppression arouses widespread investigation for the treatment of autoimmune and chronic inflammation diseases. The imbalance of Treg/Th17 cells has been proven to be closely associated with SS, owing to the regulation of the immune system. Earlier studies have revealed an increase in the pathogenic Th17 cells in patients, which contribute to the pathogenesis of SS.^11^ Since Treg cells are the main suppressors of the excessive activation of immune responses, the recent advances in the therapeutic strategy of SS are to target Treg homeostasis or its associated signaling pathway.^34^ Here we found that PTEN knockdown decreased the Treg/Th17 ratio in SPLCs, and that VIP treatment increased the Treg/Th17 ratio and the expression of PTEN. The schematic figure incorporating these signaling pathways is shown in Figure 6. Our advancements might offer a theoretical foundation for the mechanistic understanding of VIP in treating SS. Additionally, the PI3K/AKT/mTOR pathway assumes a vital role in modulating the autophagic reaction, and PTEN has pro‐autophagic activities^35^ Thus, whether VIP can protect the function of SMG cells by regulating the autophagy of the SMG epithelium is also worth paying attention to.

In conclusion, VIP exerts potential therapeutic action in SS by upregulating PTEN, acting partly through the PI3K/AKT pathway and the balance of Th17/Treg cells. Our findings may promote VIP as the potential novel SS therapeutics intervention.

## AUTHOR CONTRIBUTIONS

**Yixi Li**: Conceptualization; investigation; writing—original draft. **Wen Zhu**: Data curation; formal analysis; methodology. **Rui Lin**: Validation; visualization. **Junjie Zhao**: Resources; software. **Yue Wang**: Funding acquisition; project administration; Supervision; writing—review and editing.

## ETHICS STATEMENT

All mice care procedures and experiments were according to the ethical approval granted by the Animal Ethics Committee of Nanjing University of Chinese Medicine (No. 202101A006).

## Supporting information

Supporting information.

## Data Availability

The data that support the findings of this study are available from the corresponding author upon reasonable request.
