# Supplementary figures and images for: Vasoactive intestinal peptide exerts therapeutic action by regulating PTEN in a model of Sjögren's disease
Source: Immun Inflamm Dis. 2023 Jul 12;11(7):e936. doi: 10.1002/iid3.936 (PMC10336679; doi:10.1002/iid3.936)

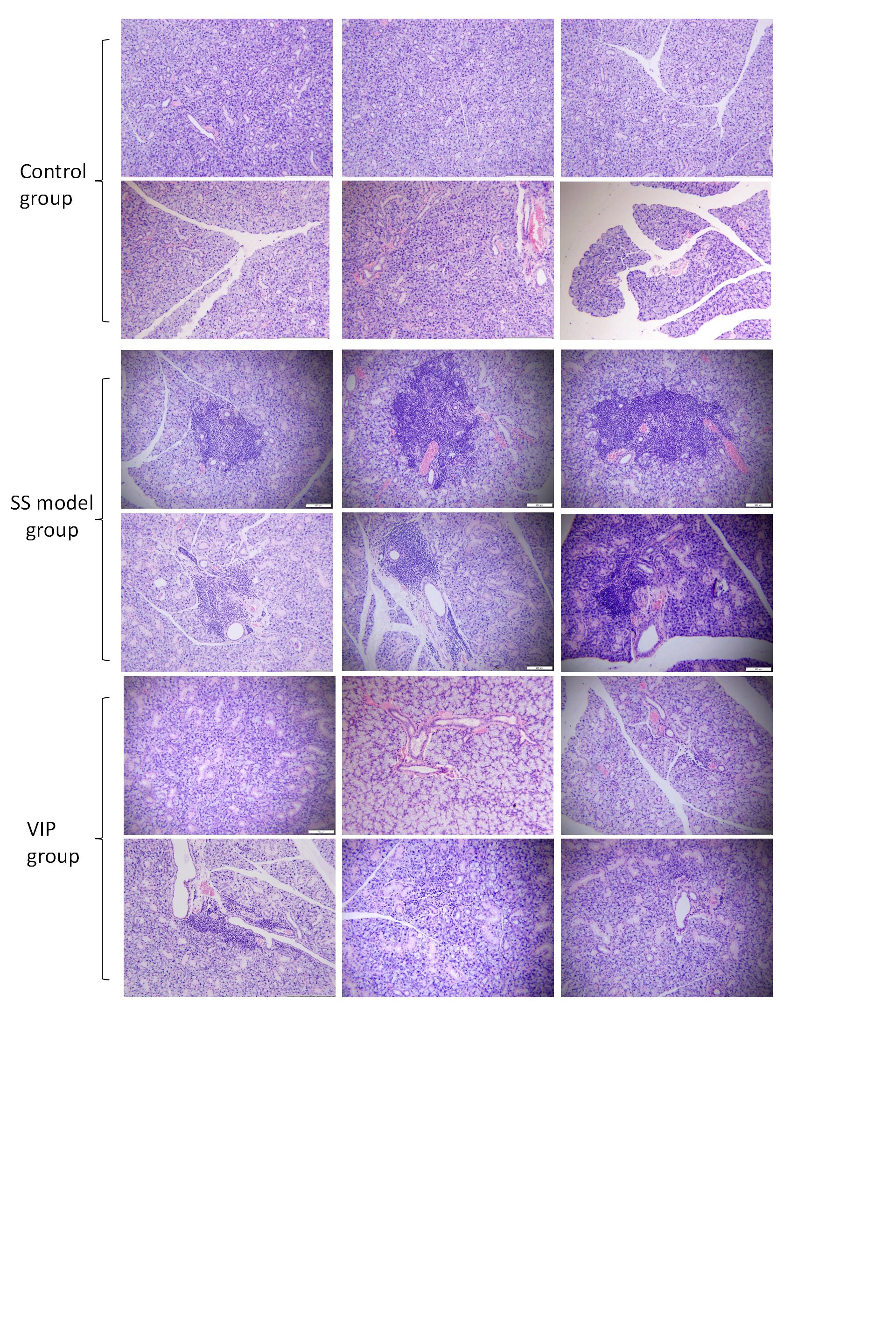

Supplement: Supplementary file 1 — Supporting information. [file IID3-11-e936-s001.jpg]
